# Nutritional status of children ages 0–5 and 5–10 years old in households headed by fisherfolks in the Philippines

**DOI:** 10.1186/s13690-018-0267-3

**Published:** 2018-04-16

**Authors:** Mario V. Capanzana, Divorah V. Aguila, Glen Melvin P. Gironella, Kristine V. Montecillo

**Affiliations:** Department of Science and Technology, Food and Nutrition Research Institute, Bicutan, Taguig City, Metro Manila Philippines

**Keywords:** Nutritional status, Young children, Schoolchildren, Fisherfolks

## Abstract

**Background:**

The study aimed to analyze the nutritional status of Filipino children ages 0–60 months (0–5.0 years old) and 61–120 months (5.08–10.0 years old) in households headed by fisherfolks.

**Methods:**

The 8th National Nutrition Survey (NNS) data collected by the Food and Nutrition Research Institute, Department of Science and Technology (FNRI-DOST) was used in the study. There were 13,423 young children and 16,398 schoolchildren participants for anthropometry component. The World Health Organization Child Growth Standards (WHO-CGS) was used to assess the nutritional status of the young children while the WHO Growth Reference 2007 was used for schoolchildren. Occupational groups were categorized based on the 1992 Philippine Standard Occupational Classification (PSOC). Descriptive statistics were used for the profiling of the different variables while bivariate analysis, logistic regression and odds ratios were used to correlate the different variables to the nutrition status of the children. Data were analyzed using Stata 12.0.

**Results:**

Results showed that households headed by fisherfolks (HHF) were one of the occupational groups with highest malnutrition among young and school-aged children. The HHF had higher prevalence of malnutrition among young children compared to the overall prevalence of malnutrition among young children in the Philippines, except for overweight. This is also true for schoolchildren, except for wasting. Age of child, sex, household size, age, fishermen and farmer as household head and type of toilet (water-sealed) were correlated to stunting, underweight, overweight and obesity among children.

**Conclusions:**

The high prevalence of stunting, underweight and wasting among young and schoolchildren in this occupational group poses immediate and serious nutrition intervention strategies such as health and nutrition information, health care, sanitation and hygiene, and physical activities. A national policy on the health, nutrition and welfare of households headed by fisherfolks and their children is highly recommended.

## Background

Fisherfolks consistently top as the poorest sector in the Philippines from 2003 to 2012 based on the report of Philippine Statistics Authority (PSA) on Poverty Incidence of Basic Sectors [1]. Aside from the uncertainty of income among fishing communities, factors such as land ownership, debt, access to health, education and financial capital, as well as political and geographical marginalization also contribute to why poverty thrives in this sector [2]. Moreover, fisherfolks often live in places that have particularly high risk of extreme events; flooding, cyclones and tsunamis often visit coastal and floodplain fisheries, while inland fisheries can be significantly affected by droughts and floods. These disasters leave severe damages on infrastructures as well as productive assets such as boat, landing sites, post-harvesting facilities and road among fishing-dependent people. These consequently decrease their harvesting capacity and access to markets, affecting both local livelihood and the overall economy [3].

These kinds of disasters had also brought severe asset damages among fishing communities in the Philippines. In 2013, Typhoon Yolanda/Haiyan, one of the strongest typhoons that visited the country, affected Eastern Samar, Samar and Leyte. The National Disaster Risk Reduction and Management Council (NDRRMC) estimated that 16 million people were affected and 1.1 million houses were damaged. The livelihood of fisherfolks was also affected in the areas. There were nearly 30,000 small-scale fishing boats damaged while more than 100,000 were lost or destroyed [4]. Additionally, about 600,000 ha of agricultural land, 33 million coconut trees and 305 km farm-to-market road were damaged, whereas more than 400 health facilities and 1200 provincial, city and municipal and barangay halls and public markets were destroyed in the area [5].

Disasters, such as typhoon Yolanda/Haiyan, can have serious consequences for food security, nutrition and health. Damaged infrastructures due to extreme events or flooding can cut access to local markets, and consequently reduce the availability of food products and increase the food prices, resulting in higher incidences of malnutrition in communities [3].

These disturbances in nutrition as a result of inadequacy in food intake, health problems, or a combination of both, invariably affect the growth of children [6]. Hence, assessments on the nutritional status of children based on their anthropometric indicators of growth has been used not only in generating information on their nutritional and health status, but also in providing an indirect measurement of the quality of life of their community, and thereby as an indicator of the nutritional status and food intake adequacy of all members in that community [7, 8].

Thus, this study aimed to focus and analyze the condition of households headed by fisherfolks (HHF) with respect to the nutritional situation by assessing the nutritional status of children ages 0–60 months (0–5.0 years old) and 61–120 months (5.08–10.0 years old), using the 8th National Nutrition Survey data. Factors affecting the nutritional status of the children in HHF were also analyzed to better understand the nutrition situation in the fishing communities in the Philippines.

The study was initiated to estimate the prevalence of malnutrition among young and school-aged children among fishing community as basis in planning and developing nutrition programs that will improve the nutritional situation of this occupational group. It is hoped that this study will provide significant information that can serve as basis for policy makers and program planners in the nutrition and fishery sectors among private or public organizations in drawing future strategies for improving the nutritional situation of the fisherfolks in the Philippines.

### Conceptual framework

**Fig. 1:**
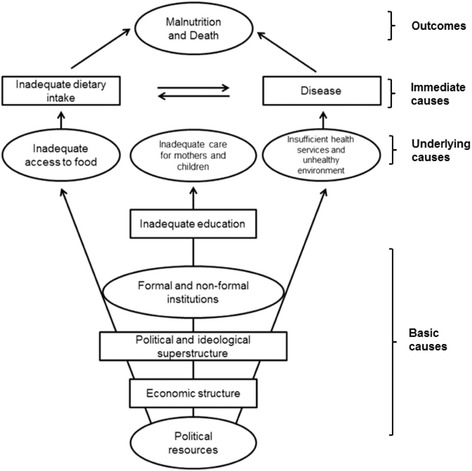
UNICEF conceptual framework for the causes of malnutrition (Adapted from Mason, 2003)

Malnutrition in children is the result of complex interaction of numerous and multifaceted factors. Thus, in the analysis of this study, the UNICEF’s conceptual framework for the causes of malnutrition was considered (Fig. 1). It was expected that factors such as age, gender, household size, occupation, and sanitation facilities were associated with the malnutrition among children in HHF.

## Methods

The study used the data from 8th National Nutrition Survey (NNS) conducted by the Food and Nutrition Research Institute, Department of Science and Technology (FNRI-DOST) in 2013. The NNS was conducted in 79 provinces, 45,047 households and 172,323 individuals, adopting the 2003 master sample developed by Philippine Statistics Authority (PSA) [9]. A stratified multi-stage sampling design for household-based surveys covering all the 17 regions, including the National Capital Region was used.

The 2013 NNS used the four subsample or replicates of the master sample for its anthropometry component. There were 13,423 young children ages 0–60 months (0–5.0 years old) and 16,398 schoolchildren ages 61–120 months (5.08–10.0 years old) participated in the survey. The World Health Organization-Child Growth Standards (WHO-CGS) [10] was used to assess the nutritional status of young children, while WHO Growth Reference 2007 [11] was used for schoolchildren.

A written informed consent was obtained from all the participants of this study through the mother or guardian. Ethical clearance was provided by the FNRI Institutional Ethics Review Committee (FIERC).

To analyze the nutrition situation of the fisherfolks in the Philippines, the households were categorized into occupational groups based on the 1992 Philippine Standard Occupational Classification (PSOC) of PSA [12]. Descriptive statistics was employed to summarize data on the prevalence of malnutrition among young children and schoolchildren in different occupational groups including the fisherfolks. Logistic regression and multinomial logistic regression analyses were used to determine the association of variables to underweight, stunting, and overweight or obesity among children 0–5 years and 6–10 years children. Statistical analysis was conducted using Stata version 12.0.

## Results

The prevalence of malnutrition among Filipino children ages 0–60 months (0–5.0 years old) by occupational group in the Philippines is summarized in Table 1. Results showed that stunting (30.3%) was the most prevalent malnutrition among children of this age group in the country, followed by underweight (19.9%). The prevalence of stunting and underweight in this age group are considered high based on the 1995 WHO cut-off for public health significance [13]. On the other hand, the prevalence of wasting among this age group was 7.9%. The prevalence of overweight was 5.0%.

**Table 1:** Prevalence of malnourished children, 0–5.0 years old (0–60 months) by occupational group: Philippines, 2013

Occupation Groups	Form of Malnutrition			
	Underweight	Stunting	Wasting	Overweight
All children	19.9 (0.4)^a^	30.3 (0.5)	7.9 (0.3)	5.0 (0.2)
• Special Occupations	17.9 (4.5)	28.1 (5.7)	3.5 (1.8)	5.0 (2.7)
• Officials, Corporate Executives, Managers and Managing Proprietors and Supervisors	13.0 (1.4)	20.2 (1.7)	6.4 (1.0)	7.1 (1.0)
• Professional	11.5 (2.5)	19.7 (2.8)	9.7 (2.3)	7.6 (2.1)
• Technicians and Associate Professionals	16.5 (2.4)	19.4 (2.5)	8.6 (1.9)	5.6 (1.4)
• Clerks	14.1 (2.6)	23.5 (2.9)	10.6 (3)	6.3 (1.8)
• Service Workers and Shop and Market Sales Workers	15.6 (1.6)	23.6 (2.0)	7.9 (1.2)	5.7 (1.1)
• Farmers, Forestry Workers and Fishermen	24.4 (0.8)	37.2 (0.9)	8.9 (0.5)	3.7 (0.3)
o *Farmers and other plant growers*	23.9 (0.9)	37.2 (1.0)	8.3 (0.5)	3.7 (0.3)
o *Animal producers*	17.5 (6.5)	24.4 (6.6)	8.4 (3.4)	5.3 (3.7)
o *Forestry and related workers*	38.7 (8.1)	44.3 (9.6)	14.2 (5.3)	9.5 (4.5)
o *Fishermen/Fisherfolks*	26.4 (1.8)	37.7 (2.0)	11.2 (1.3)	3.2 (0.6)
• Craft and Related Trades Workers	20.9 (1.3)	31 (1.5)	9.4 (0.9)	4.0 (0.6)
• Plant and Machine Operators and Assemblers	18.2 (1.2)	28.1 (1.5)	6.5 (0.7)	5.1 (0.6)
• Elementary Occupation: Laborers and Unskilled Workers	22 (1.1)	33.4 (1.2)	7.3 (0.6)	4.3 (0.5)
• No occupation	17.7 (0.9)	27.4 (1.0)	6.9 (0.6)	6.8 (0.5)

^a^Percentage (Standard Error)

The young children in household headed by fisherfolks (HHF) had higher prevalence of malnutrition compared to the overall prevalence of malnutrition among young children in the Philippines, except for overweight. The magnitude of underweight (26.4%), stunting (37.7%) and wasting (11.2%) were all alarmingly high and pose as serious public health concerns. The HHF had lower prevalence of overweight (3.2%) compared to the overall prevalence of overweight among young children in the Philippines.

Among the occupational groups, young children in HHF had the highest prevalence of underweight, stunting and wasting next to children belonging to household headed by forestry and related worker. They also had the lowest prevalence of overweight among all occupational groups.

The prevalence of malnutrition among Filipino children ages 61–120 months (5.08–10.0 years old) by occupational group in the Philippines is summarized in Table 2. Findings showed that stunting (29.9%) is the foremost form of malnutrition that is prevalent among schoolchildren in the Philippines followed by underweight (29.1%). However, unlike the observation on the young children, the prevalence of overweight (9.1%) among schoolchildren was higher compared to the prevalence of wasting (8.6%).

**Table 2:** Prevalence of malnourished children, 5.08–10 years old, by occupational group: Philippines, 2013

Occupation Groups	Form of Malnutrition			
	Underweight	Stunting	Wasting	Overweight
All children	29.1 (0.5)^a^	29.9 (0.5)	8.6 (0.3)	9.1 (0.3)
• Special Occupations	20.2 (4.5)	25.1 (5.2)	8.4 (3.7)	13.4 (3.4)
• Officials, Corporate Executives, Managers and Managing Proprietors and Supervisors	17.1 (1.4)	17.3 (1.5)	5.7 (0.8)	17.6 (1.4)
• Professional	14.7 (2.3)	14.1 (2.4)	8.5 (1.8)	22.1 (3.2)
• Technicians and Associate Professionals	20.0 (2.3)	17.9 (2.3)	7.7 (1.6)	14.8 (1.9)
• Clerks	16.2 (2.7)	16.7 (2.8)	4.6 (1.4)	18.0 (3.0)
• Service Workers and Shop and Market Sales Workers	21.8 (1.7)	21.2 (1.7)	8.1 (1.2)	11.9 (1.5)
• Farmers, Forestry Workers and Fishermen	36.6 (0.8)	40.1 (0.8)	8.4 (0.4)	4.4 (0.3)
o *Farmers and other plant growers*	35.9 (0.8)	40.1 (0.9)	8.3 (0.5)	4.5 (0.4)
o *Animal producers*	29.4 (7.6)	38.9 (8.7)	7.0 (4.1)	11.0 (5.1)
o *Forestry and related workers*	37.3 (7.5)	43.3 (7.3)	7.1 (3.0)	2.6 (2.6)
o *Fishermen/Fisherfolks*	39.9 (1.9)	39.9 (1.9)	8.6 (1.0)	4.0 (0.6)
• Craft and Related Trades Workers	31.3 (1.4)	31.4 (1.4)	10.0 (0.9)	6.6 (0.7)
• Plant and Machine Operators and Assemblers	24.8 (1.3)	23.7 (1.3)	8.0 (0.7)	11.2 (0.9)
• Elementary Occupation: Laborers and Unskilled Workers	32.9 (1.2)	33.8 (1.2)	9.9 (0.7)	5.7 (0.5)
• No occupation	25.3 (1.0)	24.1 (0.9)	9.0 (0.6)	12.9 (0.7)

^a^Percentage (Standard Error)

Among HHF, underweight (39.9%) and stunting (39.9%) were the primary forms of malnutrition among schoolchildren. Compared to other occupational groups, schoolchildren in the fishing communities had the highest prevalence of underweight. Furthermore, the prevalence of underweight among fisherfolks was even higher compared to the prevalence of underweight among all schoolchildren in the Philippines (29.1%).

The prevalence of stunting among HHF was also higher compared to the prevalence of stunting among all schoolchildren in the Philippines (29.9%). However, compared to other occupational groups, schoolchildren in HHF had the highest prevalence of stunting next to forestry and related workers (43.3%) and farmers and other plant growers (40.1%).

Moreover, there was the same magnitude of wasting among all schoolchildren in the Philippines (8.6%) and those in fishing communities (8.6%). The magnitude of wasting among schoolchildren in fishing communities was also relatively high compared to the prevalence of wasting in other occupational groups.

Fisherfolks had lower prevalence of overweight among schoolchildren (4.0%) compared to the prevalence of overweight among all schoolchildren in the Philippines (9.1%). They even had the lowest prevalence of overweight among other occupational groups, next to forestry and related workers (2.6%).

With the other variables were held constant, logistic regression indicated that both stunting and underweight were influenced by the same variables. Older children (OR = 1.03; OR = 1.11), male gender (OR = 1.15; OR = 1.08), larger household (HH) size (OR = 1.08; OR = 1.07), and the occupation of the HH head, specifically fishermen (OR = 1.36; OR = 1.44) and farmers (OR = 1.51; OR = 1.32), increases the risk for stunting and underweight respectively. While older age of the HH head (OR = 0.99; OR = 0.99) and the use of water sealed toilet (OR = 0.57; OR = 0.59) manifested a protective effect on the nutrition status. Wasting or thinness was also correlated to gender of child but was not correlated to households headed by fishermen and farmer. For overweight and obesity, older children (OR = 0.93), household size (OR = 0.96), and the occupation of the household head, fishermen (OR = 0.71) and farmer (OR = 0.77) were found to decrease its risk while the use-of water-sealed toilet (OR = 1.29), gender (OR = 1.08) and age of household head increases it. Details were shown in Table 3.

**Table 3:** Logistic regression of variables affecting the nutrition status of children (0–10 years old) of fisher folks

Variables	Odds Ratio	SE	t	*P*	95% CI	
Stunting						
Age of Child	1.03	0.005	5.47	< 0.001	1.02	1.04
Sex of Child (Male)	1.15	0.033	4.96	< 0.001	1.09	1.22
HH Size	1.08	0.007	12.06	< 0.001	1.07	1.10
Age of HH Head	0.99	0.001	−10.32	< 0.001	0.98	0.99
HH Head - Fishermen	1.36	0.089	4.75	< 0.001	1.20	1.55
HH Head – Farmer	1.51	0.057	10.79	< 0.001	1.40	1.62
HH Head – Other Occupation	base	–	–	–	–	–
Water Sealed Toilet	0.55	0.024	−13.74	< 0.001	0.50	0.60
Underweight						
Age of Child	1.11	0.01	19.92	< 0.001	1.10	1.12
Sex of Child (Male)	1.08	0.03	2.58	< 0.001	1.02	1.15
HH Size	1.07	0.01	10.69	< 0.001	1.06	1.09
Age of HH Head	0.99	0.00	−8.12	< 0.001	0.98	0.99
HH Head - Fishermen	1.44	0.10	5.46	< 0.001	1.26	1.65
HH Head – Farmer	1.32	0.05	7.36	< 0.001	1.23	1.42
HH Head – Other Occupation	base	–	–	–	–	–
Water Sealed Toilet	0.59	0.03	−12.09	< 0.001	0.54	0.64
Overweight/Obesity						
Age of Child	0.93	0.006	−11.02	< 0.001	0.92	0.94
Sex of Child (Male)	1.08	0.036	2.38	0.017	1.01	1.16
HH Size	0.96	0.007	−6.02	< 0.001	0.94	0.97
Age of HH Head	1.007	0.001	4.76	< 0.001	1.004	0.009
HH Head - Fishermen	0.71	0.045	−5.39	< 0.001	0.62	0.80
HH Head – Farmer	0.77	0.029	−6.98	< 0.001	0.71	0.83
HH Head – Other Occupation	base	–	–	–	–	–
Water Sealed Toilet	1.29	0.056	5.75	< 0.001	1.18	1.41

## Discussion

The present study showed that malnutrition is highly prevalent among children in HHF. Colds and cough, diarrhea, skin infections and asthma, sore eyes and various intestinal parasites are the common illness among children livisdng in coastal rural areas [14, 15]. These kinds of illnesses affect the growth and nutritional status of children [16]. Perhaps, the nutritional status of children in HHF was compromised due to their poor health caused by their living environment.

Another factor that affects the nutritional status of children living in the coastal areas is the high risk of extreme events, such as typhoons and tsunamis [3]. In the Philippines, many families lost their livelihood, especially the famers and fishers, when typhoon Yolanda/Haiyan visited the country. The typhoon also damaged the means of transportation (i.e. boats) of people living in far flung areas. Because of this, they experienced difficulties going to city proper or mainland where most of the evacuation areas are located and relief goods are distributed making them to go on up to four days without food. As a result, malnutrition among children increased because of difficulties in accessing nutritious foods. It has been reported that 1.5 million children were identified as at risk of acute malnutrition [17, 18].

Household socioeconomic status remains to be crucial determinant of nutritional status of children. Children in the poorest quintile had worse nutritional status than the ones from the richest group [19]. Thus, with HHF being the poorest sector in the country, the prevalence of undernutrition among fisherfolks may be due to their low economic capacity that limits their access to food and nutrition.

However, while poverty is a strong determinant of undernutrition among young children, it may not be true for schoolchildren. A study suggests that poverty is predictive factor to the poor nutrition among young children but not to the nutritional outcomes among schoolchildren [20]. On the other hand, factors such as household food insecurity, low maternal education and poor health are stronger predictor for the undernutrition of schoolchildren [21].

Furthermore, women in fishing communities often engage in economic activities to complement men’s decreasing income. In some areas, this results to substantial reduction in breast-feeding when mothers resumed their economic activities soon after delivery. Consequently, the quality of nutrition provided to the young children is compromised [22]. Moreover, various factors were also observed that are contributing to the nutritional status of schoolchildren. Quality of food intake, food availability, household size, literacy of person in charge of food preparation and household head are some of the factors associated to the nutritional status of schoolchildren. Food availability and nutrition education on balanced diet, food production and consumption are necessary, although not sufficient, to improve the nutritional status of schoolchildren [23].

Perhaps, in the case of fisherfolk communities in the Philippines, the living condition of this occupational group coupled with poor health, high risk to extreme events, poverty and poor quality of diet may have contributed to the occurrence of various forms of undernutrition among young and school-aged children.

On the other hand, along with high prevalence of undernutrition, overnutrition was also observed among young children in fishing community in the Philippines. Generally, overweight among young children is usually attributed to excessive calorie consumption and low calorie expenditure. The increased consumption of more energy-dense, nutrient-poor foods with high levels of sugar and saturated fats, together with reduced physical activity, have led to obesity rates that have increased three-fold or more since 1980 [24]. However, these reasons may be unlikely in poor households. A study suggests that overweight among young children in food insecure households may be due to other potential factors linked to obesity, like low activity levels and excessive television watching [25].

Moreover, overweight among children observed in coastal areas may be due to geographical disparities in terms of income. One study observed that those in coastal area with high economic status exhibited increase in the prevalence of overweight and obesity than those in other areas with less economic development [26]. Perhaps, in the present study, the overweight children in HHF may be located in coastal areas with high economic status.

Looking into the prevalence of malnutrition among children in HHF, it can be observed that there is a low prevalence of overweight and high prevalence of underweight, stunting and wasting children. This scenario can be attributed to limited access of children to high-calorie snacks and fast food which are hardly affordable. Thus, there are only few who are identified to overweight. Another plausible explanation given is that the children may have been engaged in a more physically demanding activities compared to their contemporaries [27].

Based on the results of the present study, there is a need for immediate action to intervene in the magnitude of undernutrition among this occupational group with priority on the needs of children. Young children are more prone to poor nutrition and health conditions than adults. Poor nutrition among children of this age could not only contribute to increased likelihood of contracting serious illnesses but may also have a permanent effect on their health and development [28].

In addition, aside from the direct welfare and financial costs of illness due to poor nutrition, schoolchildren with poor health can also grow up as an adult with poor lower health status with less education which in turn could result to intergenerational transmission of poverty [29]. Childhood undernutrition exposure has permanent socioeconomic and health consequences. These consequences may include metabolic and cardiovascular diseases, reduced learning ability, lowered years of schooling and intellectual performance in adulthood, reduced working ability, productivity and income, poor quality of life and overall poverty that could be transferred to the future generations [22].

Thus, investing in the nutritional status of young children and schoolchildren in HHF could break the cycle of poverty in this occupational group. Alleviating the poverty has been a challenge for the longest time now and starting from the poorest of the poor sectors in the country should be a good start.

## Conclusion

Findings showed that young children in HHF had higher prevalence of malnutrition compared to the overall prevalence of malnutrition among young children in the Philippines, except for overweight. This is also true for schoolchildren in HHF, except for wasting. It also identified that fisherfolks were one of the occupational groups with highest malnutrition among young and school-aged children. The high magnitude of public health concern in terms of underweight, stunting and wasting among these age groups pose an immediate and serious need for action, prioritizing the nutritional needs of this occupational group.

Various factors could affect the nutritional status of children in fishing communities. Food security, socio-economic capacity, availability of health services, environmental sanitation and geographical disparity in terms of economic development are some of the factors that should be considered in formulating and prioritizing in nutrition programs for children in this occupational group. Possible interventions should include health and nutrition education program that advocates the promotion of children’s nutrition at home, physical activity, capacity building, sanitation and hygiene in the community. A national focus on the health, nutrition and welfare of fisherfolks is highly recommended.

Furthermore, forestry and related workers is another occupational group that exhibited alarmingly high prevalence of malnutrition among young and school-aged children. Perhaps, for future studies, it would be an interesting topic to explore and understand the determining factors of malnutrition in this occupation group.

## Abbreviations

APIS: Annual Poverty Indicators Survey
FIERC: FNRI Institutional Ethics Review Committee
FNRI-DOST: Food and Nutrition Research Institute, Department of Science and Technology
HHF: households headed by fisherfolks
NDRRMC: National Disaster Risk Reduction and Management Council
NNS: National Nutrition Survey
PSA: Philippine Statistics Authority
PSOC: Philippine Standard Occupational Classification
WHO-CGS: World Health Organization-Child Growth Standards
